# Computational Framework to Model the Selective Laser Sintering Process

**DOI:** 10.3390/ma17081845

**Published:** 2024-04-17

**Authors:** João Castro, João Miguel Nóbrega, Ricardo Costa

**Affiliations:** Institute for Polymers and Composites, University of Minho, Campus de Azurém, 4800-058 Guimarães, Portugal; rcosta@dep.uminho.pt

**Keywords:** SLS, polymers, particle scale, computational modeling, OpenFOAM

## Abstract

Selective laser sintering (SLS) is one of the most well-regarded additive manufacturing (AM) sub-processes, whose popularity has been increasing among numerous critical and demanding industries due to its capabilities, mainly manufacturing parts with highly complex geometries and desirable mechanical properties, with potential to replace other, more expensive, conventional processes. However, due to its various underlying multi-physics phenomena, the intrinsic complexity of the SLS process often hampers its industrial implementation. Such limitation has motivated academic interest in obtaining better insights into the process to optimize it and attain the required standards. In that regard, the usual experimental optimization methods are time-consuming and expensive and can fail to provide the optimal configurations, leading researchers to resort to computational modeling to better understand the process. The main objective of the present work is to develop a computational model capable of simulating the SLS process for polymeric applications, within an open-source framework, at a particle-length scale to assess the main process parameters’ impact. Following previous developments, virgin and used polymer granules with different viscosities are implemented to better represent the actual process feedstock. The results obtained agree with the available experimental data, leading to a powerful tool to study, in greater detail, the SLS process and its physical parameters and material properties, contributing to its optimization.

## 1. Introduction

### 1.1. Selective Laser Sintering

**Figure 1:**
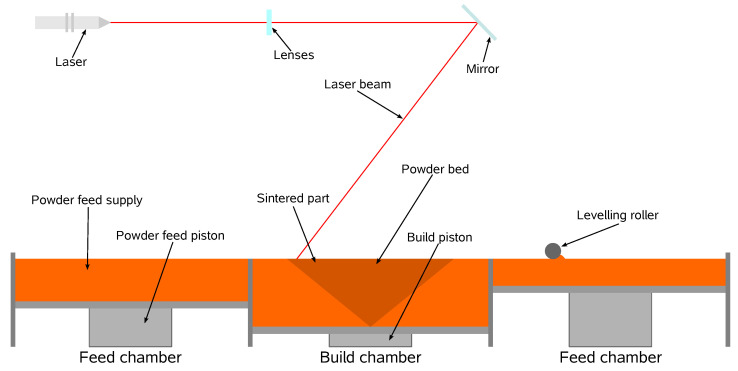
General representation of the selective laser sintering (SLS) process (adapted from [4]).

Selective laser sintering (SLS) is a powder bed fusion (PBF) sub-process, which, according to the ASTM technical committee [1], is one of the seven leading additive manufacturing (AM) processes. In SLS, parts are built layer-by-layer, enabling the manufacture of highly complex geometries, eliminating the need for extra tooling and reducing material waste since only the consolidated material is used. Such configurations, when coupled with quickly iterative designs, make the process more cost-effective and ideal for prototyping and low-quantity productions. Compared to other AM processes, SLS stands out by using a high-density energy source, the laser, which, when paired with the powder bed particles’ small size, offers higher resolution, accuracy, and surface finish quality [2]. That, coupled with better mechanical properties, faster printing speeds and its versatility in machine configurations and compatible materials, makes SLS the most popular AM process amongst engineering applications [2]. A general representation of the SLS process is illustrated in Figure 1. SLS machines are divided into two zones: the build chamber, where the parts are built, and the feed chamber, which stores the necessary powdered material for the process. Despite the laser being powerful enough to melt the material at room temperature, the powder and chamber atmosphere is pre-heated to a temperature just below the material’s melting point. The pre-heating phase, besides minimizing the laser power requirements and allowing faster scanning speeds, reduces the temperature gradient between scanned and non-scanned areas, keeping the overall temperature more uniform, not only facilitating the coalescence between layers, but also helping to minimize thermal originated defects caused by non-uniform thermal expansions and contractions, such as local shrinkage and thermal distortions [3]. This phase is conducted in bulk by heating elements located all around the system, affecting all of its constituents, and it is maintained throughout the entire building phase. Because of the material’s prolonged exposure to high temperatures, the process occurs inside a closed chamber filled with an inert gas to reduce its degradation. After pre-heating, the process starts with spreading the first powder layer, with the build piston in its highest position, followed by the leveling roller movement that spreads the material from the feed chamber to the powder bed inside the build chamber. After the layer completion, the laser beam is directed to the material in the powder bed through a lens and guided by moving mirrors. The material scanned by the laser receives enough energy to melt and coalesce with adjacent particles, including the ones on the previous layer. SLS does not require support structures since the non-melted particles stay in place, supporting the subsequent layers. This feature reduces material waste and facilitates building multiple parts in the same bed since the space otherwise occupied by support structures is now free. Following the scan of the present layer, the build piston lowers itself and, consequently, the powder bed by the thickness of one layer and a new layer is spread on top. From this point, the described operations are repeated until the desired part is built and, at that instant, the heating phase is terminated and the part is left to cool inside the controlled machine chamber to promote uniform cooling in an inert atmosphere. After cooling, the built parts are removed and, if necessary, subjected to finishing operations.

### 1.2. State of the Art

The SLS process’s current state already allows for manufacturing various parts with highly complex geometries and, in some cases, with notable mechanical properties [5]. However, many defects still need to be solved to allow the process to meet industry requirements and evolve from a prototyping process. The process optimization is typically performed based on trial-and-error experimental approaches, which are expensive, time-consuming and often fail to provide detailed information and optimal configurations. Such conditions highlight the need for better alternatives, such as computational modeling, largely used to simulate various technical processes with similarly complex natures to the SLS process. Modeling SLS processes poses several challenges because of its multi-disciplinary nature [6]. For example, there are several interactions between the laser and the material, such as absorption and reflection, as well as the subsequent heat transfer and phase transformation that, due to fluid flow, requires a moving interface between phases. Also, there are non-linearities, mainly the temperature-dependent material properties. Moreover, as usual, the problem must be spatially and temporally discretized. However, if the model scale is small enough to consider the individual particles to account for powder-related defects, it will require high mesh resolutions and small time scales, demanding significant computational power. Given the high computational requirements, it is relevant to investigate the impact of each process phenomenon and whether simpler models can provide valuable insights about each parameter. Unlike conventional polymer manufacturing processes thoroughly studied in the past decades, SLS optimization with computational modeling is relatively recent and only a few works can be found [7]. Due to the associated complexity, different numerical approaches are employed to simulate each process step based on their suitability for the task, a factor that has limited researchers who need to integrate various methods. For example, the discrete elements method (DEM), a mesh-free method, is often used to simulate the powder bed deposition step because of its particle simulation nature that allows accounting for the interactions between particles [8]. Similar approaches attempted to extend this method to include other interactions, such as heat transfer, to predict the powder bed temperature distribution [9,10]. Unfortunately, the nature of the DEM does not support fluid flow and treats particles as a single undiscretized element with uniform temperature, significantly impacting the result’s accuracy. Conversely, although not ideal for particle simulation, mesh-based methods are more suitable for simulating other process steps. For example, the finite element method (FEM) has been extensively used to simulate the SLS process [11,12,13] due to its simplicity, efficiency and the vast available literature on the topic. The finite volume method (FVM) is also employed because of its conservative nature, helping to surpass some limitations present in the FEM [14]. Since mesh-free and mesh-based methods present distinct advantages, some researchers couple them to simulate separate steps [15,16].

When metals are employed, the process is known as selective laser melting (SLM), in which case different models are required for the powdered and liquid state due to their distinct properties [16,17]. Indeed, most of the literature is dedicated to the SLM process, whereas the SLS is not so well developed. Although some differences arise in terms of modeling according to the material employed, both processes share common principles and objectives. In that regard, most of the research on the topic shares the objective of developing a model that accounts for various process parameters, aiming at assessing their influence to optimize the process. However, not all studies follow the same strategies or assumptions. Considering the powder bed as a continuous media, Bugeda et al. [18] developed a 3D model to simulate the sintering of a single track, obtaining the temperature profiles and liquid fraction. Later, other authors assessed the influence of some process parameters, such as laser and scanning speed [19] and scanning pattern and speed [20]. Dong et al. [21] developed a model that included temperature-dependent material properties to simulate the transient heat transfer and obtain the temperature and density distributions. Aiming at predicting the fusion depth, Riedlbauer et al. [22] changed the scanning parameters in a model developed with a more realistic heat source. Peyre et al. [23] and Foroozmehr et al. [24] conducted similar studies to assess not only the same parameters effect but also the optical penetration depth, coupling the results with experimental validation. More recently, Mokrane et al. [14] developed a complex model that included multiple layer deposition to evaluate the heat transfer between them and the resulting densification/shrinkage, and Shen et al. [25] considered the crystallization and residual stresses, but only for one layer. Li et al. [26] performed a much longer time-scale simulation to observe the temperature profiles from the pre-heating to the cooling phases. On a different note, Kim et al. [27] developed a deep learning model to calculate the best scanning path solely based on temperature profiles. For further literature review, the interested reader is referred to some recent reviews on the topic [17,28,29,30,31].

Most simulation studies on the SLS process are based on an unrealistic powder represented by a continuous media with mixed properties. However, some authors have already tried to surpass this limitation, such as Osmanlic et al. [32], who developed a 2D model with a very accurate laser source that accounted for reflection, refraction and attenuation used on a non-real geometry with different sized granules, aiming at determining the bed density influence on the scan effect. Considering an arbitrary powder distribution, Bierwish et al. [33] went a step further to visualize the coalescence development after the scan and the scan parameters’ influence on it. Zhang et al. [34] progressed to a 3D simulation with a much more representative powder bed and simulated the sintering evolution after the scan with DEM, but, as mentioned previously, the results were limited by the selected method nature. Recently, the authors of the present work, Castro et al. [4,35], developed an open-source coupled DEM–FVM approachm where the most critical process parameters influence was evaluated on a realistic powder bed. The developed computational framework couples a DEM approach for the powder bed formation simulation and uses the resulting geometry for a CFD simulation on an FVM software. It is, therefore, more powerful and complete than the conventional approaches, allowing a more comprehensive and representative simulation of the process. With this method, critical phenomena, such as the sintering evolution, are visible at the particle scale, and several defects related to it are observed alongside each process parameter’s influence on it. Despite the significant advances, some limitations remain to be addressed, which the current publication aims to surpass.

### 1.3. Objectives and Work Outline

The current work is a continuation of an ambitious project that aims at developing a solver within an open-source framework capable of accurately simulating the SLS process at the particle length scale to assess its main process parameter’s influence. In the previous publication, a set of tools was used for that purpose and a significant advancement in the SLS process’s current state-of-the-art was achieved, with the current developments comprising an approach where the most prominent process parameters are considered and their influence evaluated at the micro scale. However, the obtained results are limited to energy-related conclusions because of the solver’s immiscible nature, which does not allow for different liquids with distinct viscosities to coalesce. This was considered a major limitation since the virgin and used particle viscosities are so distant and result in contrasting coalescence rates that need to be represented individually. Therefore, this paper is focused on describing the steps taken to overcome the identified limitation, as well as using the improved tool to perform several case studies, aiming at better understanding the process parameter’s influence while trying to propose optimizations for the process itself and compare the results with experimental data to validate the developed framework. For that purpose, this journey starts with a computational framework familiarization, in Section 2, where the reader can briefly become accustomed to the work developed up until the current point. Then, in Section 3, the current limitations are presented in detail alongside a thorough discussion on the importance of overcoming them, and the tool modification is proposed, explained and illustrated. Finally, with the improved tool, several case studies with various aims are presented in Section 4.

## 2. Computational Framework

In this section, the previously developed computational framework is briefly described. Extensive research was conducted prior to utilizing the mentioned tools to evaluate their suitability and validate their findings. Since the current paper primarily aims to highlight recent advancements, readers are directed to previous publications for comprehensive information on the selection and assessment of the mentioned tools [4,35].

### 2.1. Power Bed Formation

**Figure 2:**
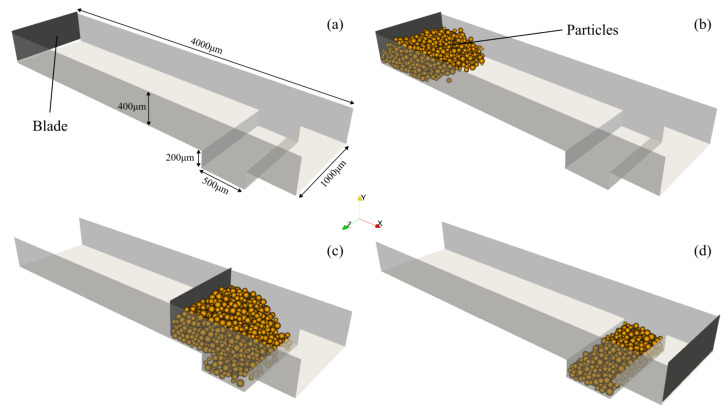
Powder bed formation simulation. (**a**) Geometry dimensions; (**b**) particle insertion; (**c**) blade movement; (**d**) final state (adapted from [4]).

The powder bed formation simulation’s aim was to obtain a representative geometry in terms of particle size and size distribution. For that purpose, following the work’s open-source nature, LIGGGHTS [36], a DEM particle simulation software was used to generate a representative powder bed section to perform the subsequent simulation studies. Due to the particle’s typical small size, simulating the power bed formation for the whole build chamber would require considerable computational resources in terms of both time and memory, limiting the studies to be performed, which motivated the initial use of only a small representative section of the powder bed. However, by employing appropriate models, validated in the literature, and accurate material data in the simulation, the resulting apparent density for the representative section was 0.43 g/cm^3^. This value is very close to the experimental value of 0.45 /cm^3^ [37], thus demonstrating the suitability of the simulation for analyzing the SLS process at the desired particle length scale, despite the simplifications made. The powder bed formation simulation consists of a box and a moving blade, both illustrated in Figure 2a. Initially, the particles, with a realistic particle size distribution, are inserted into the domain (Figure 2b), and then the blade moves, dragging them along (Figure 2c), until they fall inside the desired box and the excess particles are deleted (Figure 2d). That box limits the representative section which spans, vertically, approximately two material layers.

### 2.2. Sintering Simulation

The selected software for the sintering simulations was OpenFOAM [38], a widely employed open-source library to solve complex computational modeling problems. From its library, the icoReactingMultiphaseInterFoam solver, originally released with OpenFOAM version 1806 [39], was selected since it meets the SLS process modeling requirements, namely its multiphase nature and also being capable of representing the most relevant sintering mechanisms. The solver also allows the selection of a thermodynamic model for each phase, accounts for surface tension effects and supports mass and heat transfer between different phases. Lastly, it contains a volumetric heat source laser radiation model that follows a Gaussian distribution, which attenuates as it passes through the material. In a previous study [4,35], each model was examined and evaluated alongside the solver itself to confirm their suitability and stability. The impact of the mesh refinement level was assessed and the chosen one was used in subsequent studies, including the following. Furthermore, the most significant model studies and the solver’s overall ability to simulate the SLS process were depicted, from which some results are reproduced below to illustrate the current limitations.

### 2.3. Solver Assessments

**Figure 3:**
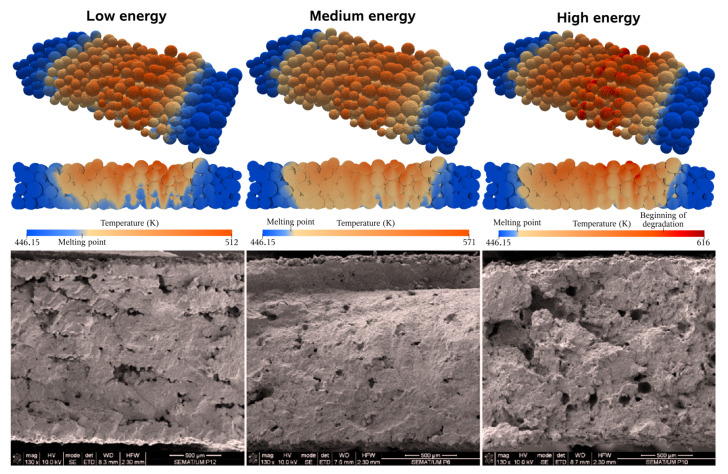
Assessment studies comparing simulation results (**top** row) with experimental ones (**bottom** row). From left to right, low-, medium- and high-energy cases. (Adapted from [4,40]).

The models available in the icoReactingMultiphaseInterFoam solver were qualitatively tested and validated against experimental data. The experimental assessments performed in Lopes et al. [40] were used as a reference, which includes three cases differing only on the laser power, as illustrated in Figure 3. All of the models were compared with simulation results after the laser scan by analyzing the induced temperature maps and correlating them with the identified defects. For example, in the high-energy case (37 W), it was experimentally observed that some zones suffered thermal degradation. The corresponding simulation temperature profiles show similar results, with some zones surpassing the material degradation temperature, proving that partial degradation is expected to occur. For the medium-energy case (30 W), a dense part with developed coalescence and minor porosity was obtained in the experiments. The corresponding simulation results in high-temperature zones, which increases the material mobility but is below the degradation point, thus indicating that the coalescence should be enhanced. In the low-energy case (17.1 W), the porosity is accentuated due to a lack of coalescence in some regions. Based on the counterpart simulation results, one can conclude that the much lower overall temperatures greatly hindered the material mobility by exponentially increasing its viscosity, resulting in poor coalescence, especially between layers. To conclude, the simulation results, based on temperature profiles alone and the information they provided, along with the extrapolated details from it, correlated positively with the experimental observations, which allowed drawing several important conclusions on the solver’s accuracy and advancing with more elaborated studies.

## 3. Solver Improvement

### 3.1. Current Limitations

**Figure 4:**
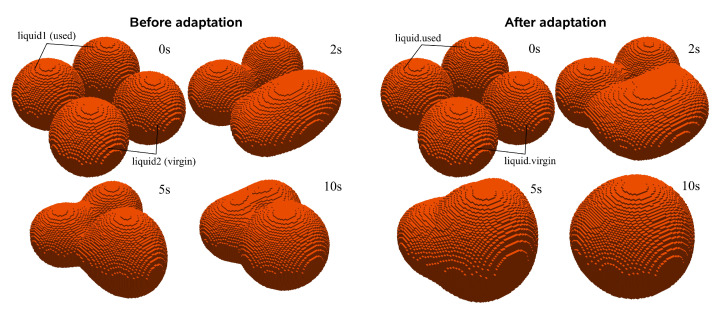
Comparison between the coalescence behavior before (**left** image) and after (**right** image) the code adaptation, demonstrating the multiple viscosity implementation.

As observed in the previous section, the current developments consist of a solver capable of simulating the SLS process and assessing the influence of its main parameters on a realistic geometry. Despite the usefulness of such results, due to the solver’s immiscible nature, it limits them to only addressing thermal effects. This restriction renders them insufficient because they cannot account for the sintering evolution, which is an important phenomenon expected to influence the outcome. Indeed, several SLS problems are related to this evolution, most predominantly with distinct coalescence developments caused by different material viscosity levels, which arise from the inevitable powder reusing [41]. Unfortunately, despite the necessary models to describe the sintering evolution being available in the OpenFOAM library, two liquids of the same material with different viscosity values (the virgin and used material liquid phases) cannot coalesce. Indeed, the current computational model assumes that phases having different properties stand for different immiscible liquids while, in practice, coalescence is still observed. This limitation is better illustrated in Figure 4, where two pairs of spheres, each pair belonging to different liquid phases, are in contact and suspended in the air without gravity. One liquid phase stands for the used material, while the other represents the virgin material. As observed, especially for the virgin phase, which has much more mobility, due to its lower viscosity, coalescence only occurs between the same liquid phases. After ten seconds, the virgin phase spheres have merged into a single sphere, but coalescence between the different phases does not occur. This behavior is expected according to the implemented immiscible assumptions of the employed solver but is physically incorrect when compared with the actual sintering evolution in the SLS process, heavily limiting its potential.

One could assume that since the solver is capable of describing the sintering evolution for a single liquid phase, a possible solution would be to select an average viscosity value for the particles. This appears to be a reasonable simplification since the SLS process is already often depicted with the assumption of intermediate values for the material properties; however, such an assumption is far too simplified to allow any acceptable conclusions. In fact, this approach was explored in the preceding work [4], when surface tension studies were performed for the different viscosities. It was observed that, for a very simplified case involving only two spheres, selecting the average viscosity value between the virgin and used phases would lead to complete coalescence (if the cases were extended long enough) within the timeframe that falls between the individual requirements for the virgin and used materials. This was expected because both the viscosity and surface tension are constant values that behave as opposing forces. At the time, because a viscosity value was required to initiate the simulations, the average value was selected, but the extended cases were purposely not exhibited, because it could not represent the system behavior. In fact, the cases were extended during the solver exploration phase [35] prior to the published article, and during the comparison between the average and used viscosity values, it was concluded that “because the surface tension is modeled as a constant and the values are the same for both cases, the results are almost identical, but slower...”. The employed viscosity values of 390 and 5095 Pa· s for the virgin and used materials, respectively, are too far apart to be simplified to a single value and, besides that, the presence of the distinct values is the base for a good representation. Analyzing Figure 4 again, it is clear that the different phases evolve at distinct rates, for example, it takes the used liquid to be at the same state of coalescence as the virgin after just two seconds, which is five times slower. These contrasting coalescence rates indicate that, while one phase hardly moves, the other has already surrounded various particles and slipped through the voids, promoting good adhesion inside the layer. This opposing behavior was also observed and discussed in experimental settings, as illustrated in Figure 3, and is the main reason to look for a more realistic model.

### 3.2. Modified Solver

The employed solver was conveniently modified to overcome the identified limitations, while the overall features and immiscible assumption are preserved. The approach resorts to an existing feature, originally implemented for chemical reactions that allow multi-species liquids [39]. In that case, a single phase can comprise multiple sub-phases that transport different chemical species, each having specific thermophysical properties. With this feature, the polymer liquid phase could comprise two distinct sub-phases, representing the virgin and used materials. Although neither of the sub-phases actually contain different species to transport, this approach allows the prescription of different viscosity values for each sub-phase and effectively circumvents the coalescence limitation since both species belong to the same liquid. Unfortunately, the phase transition from solids to different multi-species liquids, despite being allowed in the solver, is a feature that was implemented without considering the possibility of selecting the species that the solid is transforming into, and the phase fraction values are incorrectly distributed across each existing species. Among this, there are other less relevant inconveniences in the solver, such as not allowing temperature-dependent multi-species liquid phases to exist in the first place, which were easily surpassed without major alterations.

**Figure 5:**
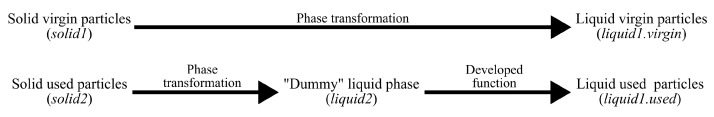
Schematic representation of the implemented code approach to surpass the identified limitation.

Aiming at modifying the solver to fulfill the presented needs, the algorithm logic was adapted to work with a specific setup, which is illustrated in Figure 5. Initially, a set of particles is defined as solid *solid1* and the remaining as solid *solid2*. Although unnecessary, both contain the same properties, and their segregation is only used to distinguish the particles of used and virgin material. When receiving enough energy for phase transition, *solid1* phase changes into *liquid1*, with species set as virgin, which denotes it as *liquid1.virgin*. On the other hand, due to the previously mentioned species phase change limitation, *solid2* phase changes first into *liquid2*, a simple, single-species liquid phase, and only after into *liquid1.used*. *liquid1* is a multispecies liquid phase whose two species hold the used and virgin materials, required such that *liquid1.used* and *liquid1.virgin* can coalesce. For that last transformation, a function that forces *liquid2* to transform into *liquid1.used* after the phase transition, was implemented inside the species equation file [42]. This function takes action immediately after the function that is responsible for calculating the mass exchange for the species, consistent with the previously computed phases’ source terms, which happens right before the function that solves for the species. With this strategy, the multi-species phase change limitation is overcome by adding a “dummy” phase (*liquid2*) that temporarily stores the information before passing it to the intended phase (*liquid1.used*). The proposed modification should not cause any implications on the solver’s performance or reliability and its stability and computational cost remain unchallenged because no additional features are being added but instead a way of allowing existing features to perform according to the needs of the work. The computational time does increase when compared to the average viscosity implementation, not due to the implementation itself, but because the problem is limited by the Courant number, with a maximum value of 1, and an increase in material mobility, which results from the low viscosity phase and causes higher velocities, which, in turn, forces the time step to be lower. Also, this further proves the new implementation isolation in the original code since the modification is performed in the species equation, the remaining variables are unaffected, and their calculations are uncompromised.

For verification purposes, the same test case, regarding the coalescence between two pairs of spheres, is performed with the proposed implementation, and the results are illustrated in Figure 4. Besides observing coalescence between spheres with the same viscosity value, coalescence between spheres with different viscosity values is now evident. Indeed, the low viscosity of the virgin spheres compensates for the lack of mobility of the used spheres, evolving into their lowest energy state of a single larger sphere. Although the proposed implementation is case-specific for the SLS process, it allowed us to effectively overcome the coalescence limitation between virgin and used materials in the original code.

## 4. Case Studies

With the proposed implementation, several studies were conducted to assess the solver sensitivity to the most relevant process physical parameters, namely the hatch distance, the laser power and the scan speed. For these studies, unless specified otherwise, the considered parameters are those reported in Table 1, which were selected in a previous work phase [4]. The process parameters are from the low energy case, which is the reference for the following studies for several reasons, primarily because, out of all the experimental studies, it was the single case where the computational domain, in thickness, was not fully melted, making it the only one where it is possible to observe alterations in fusion depth in both directions. Moreover, no other experimental case showed major signs of poor coalescence due to high viscosities, which is what is trying to be observed. Last, but certainly not least, the reached temperature during the evolution phase is, on average, close to the one for the material experimental data used, therefore, the viscosities should be similar and the sintering incomparable.

### 4.1. Hatch Distance

**Figure 6:**
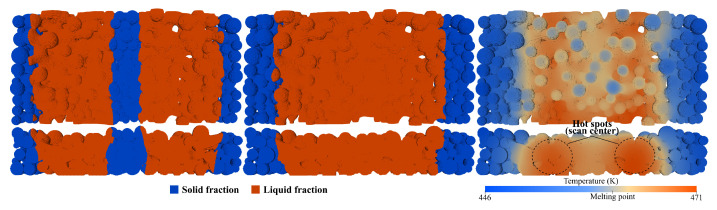
Hatch distance studies for the highest (**left** image) and lowest (**middle** image) values, with the temperature profiles for the latter (**right** image).

The experimentally selected hatch distance between scan paths was 0.3 mm, which suited the quality requirements of the studies. Increasing the hatch distance can, however, improve the SLS process efficiency by making it cheaper since fewer laser scans require less energy and time. In that regard, in addition to 0.3 mm, hatch distances of its maximum theoretical value (0.4734 mm), where both scans are adjacent, and an intermediate value between those two (0.3867 mm), are considered. Figure 6 shows the simulation results after two seconds, where the liquid fraction increase has reached its maximum and is highlighted as orange. The first image (Figure 6 left) shows the results obtained for the maximum hatch distance, where a significant portion of unmelted material separates the adjacent scans, clearly indicating that this value is excessive. However, in the second image (Figure 6 middle), this separation disappears, and the two scan portions are now connected. However, it is still unsafe to assume this value is acceptable without further investigating the temperature profile at this instant, which is presented in the right image (Figure 6 right). Despite the overlap scanned material being effectively fused, its temperature is very low, almost the same as the melting point. That is further proved by two hot spots in the center of both scans, clearly separated by the overlapped zone that melted through heat diffusion. Overall, it is known that higher temperatures promote previous layer remelting, which is essential for proper layer adhesion, and lower temperatures hinder material mobility, leading to several defects. Also, the non-uniform temperatures along the center might induce cooling-related problems, such as warping, caused by different contraction levels. Nonetheless, if only the liquid fraction is considered, with the current energy parameters, increasing the hatch distance from 0.3 mm to 0.3867 mm will result in faster process times.

### 4.2. Laser Power

**Figure 7:**
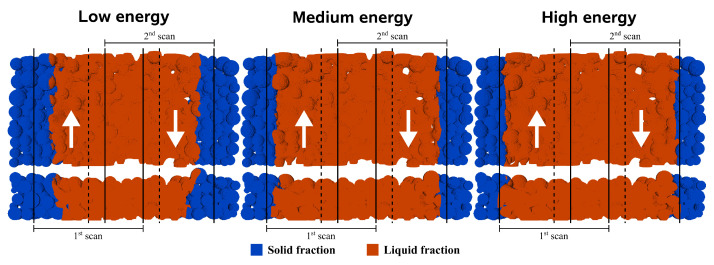
Laser power influence on scan area studies for the low-, medium- and high-energy cases.

The dimensional accuracy is essential to evaluate in order to improve the process capabilities. Assuming that the part shrinkage is uniform, the accuracy depends only on the melted material portion, which is dependent on the energy parameters and consequent lateral heat conduction. Due to the polymer’s typically poor thermal conductivity and the laser’s Gaussian energy profile, the scan limit might not coincide with the melted area limit, and a scan offset is required to compensate for that deviation. Three cases were prepared to study the laser power impact on the melted area. The results are displayed in Figure 7, which have the laser power values of the assessment study cases of 17.1 W, 30 W, and 37 W, from left to right, respectively. The arrows indicate the scan direction, the dashed lines represent the scan path center and the continuous lines its limits, with the liquid fraction displayed as orange two seconds after the scan, when its value is maximum. For the lowest power, as expected, the fraction of unmelted material relative to the scanned area is accentuated. Increasing the power to 30 W significantly increased the melted area, but still not enough to fill the scanned area. Surprisingly, even with the high-energy case parameters, where no more energy can be introduced because there is already substantial degradation, the scanned area is still partially unmelted. One can conclude that the Gaussian energy profile, coupled with the material’s low thermal conductivity, limits the lateral energy supply and dissipation. These results prove that the computational approach is useful for adjusting SLS machine software to correlate the scanned and melted areas better.

### 4.3. Energy Density Studies

**Figure 8:**
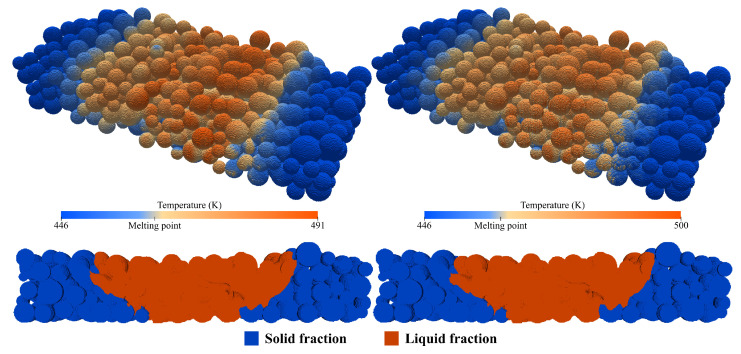
Energy density studies for the same energy density, altering only the laser power (**left** image) or scan speed (**right** image).

Most experimental works rely on energy density to optimize the process, namely on Drexler’s equation [43], because it provides a practical and simple relation between the most relevant process parameters and is given as:

(1) $$E_{D}=\frac{P_{L}}{v_{s}H_{s}d_{layer}},$$

where $E_{D}$ is the energy density, $P_{L}$ the laser power, $v_{s}$ the scan velocity, $H_{s}$ the hatch distance and $d_{layer}$ the layer thickness. The following studies present two objectives: firstly, determine the lowest energy limit or, in other words, the parameters that result in the fusion of a single layer (half the computational domain) and, secondly, assess the Drexler’s equation accuracy by changing the laser power and scan speed, while maintaining the same energy density ($E_{D}$). The results of these case studies are illustrated in Figure 8. On the left, by lowering the power to 10 W, the fusion of approximately a single layer was achieved. The fusion depth in the domain’s center portion is superior to one layer due to the laser Gaussian energy profile that concentrates the energy in the center, which is why it is impossible to make the scan extremities’ depth match the middle ones. Considering that, the value of 10 W was assumed to be the lowest because the extremities represent, on average, one layer, making this set of parameters the lowest energy limit. Finding this limit could be useful for specific builds where additional energy is not required to reduce the viscosity further. Using Drexler’s equation, the $E_{D}$ for this limit was calculated, and then, to maintain it, instead of lowering the laser power, the scan speed was increased from 3 m/s to 5.13 m/s. The results, illustrated in the right image, show very similar temperature profiles to the previous case, with a maximum temperature just a few degrees above. This temperature increase might be related to the lesser time given for the energy to diffuse since the liquid fractions, highlighted as orange two seconds after the scan, are indistinguishable. Based on these results, it is safe to assume that the laser power and scan speed can be changed for a certain energy density value.

### 4.4. Coalescence Development

**Figure 9:**
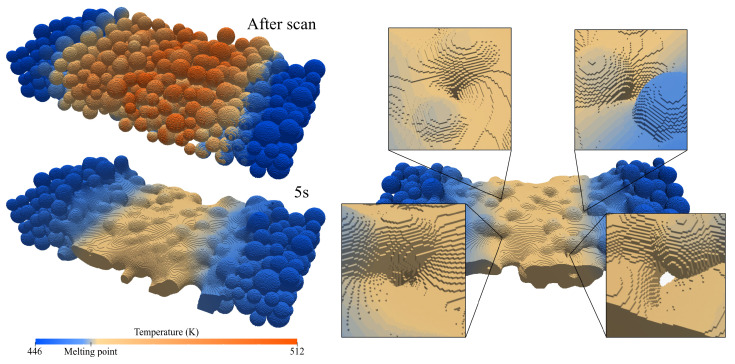
Coalescence development studies showing the sintering evolution after five seconds and highlighting defects related to it.

One of the most relevant challenges associated with the SLS process is the insufficient coalescence that may lead to porosity or overall poor adhesion between particles, as observed in the experimental reference case. This defect directly results from material reusing, substantially increasing its viscosity. To better understand the coalescence development, the base case was run for a more extended period of five seconds, and the results are shown in Figure 9. The first and most obvious observation is that layer densification is mainly caused by the coalescence between the particles, significantly reducing porosity and, consequently, layer height. Gravity also influences height reduction, but considering the high viscosity and reduced mobility, the sintering forces are much more relevant. Also evident is the used particles maintaining their shape almost entirely, which leads to the expected conclusion that the observed overall good coalescence exists thanks to the low viscosity fluid that binds all the particles together. Then, in the right part of the figure, some zones resembling the typical experimental defects are highlighted. In these four zones, used particles were adjacent to each other, without lower viscosity virgin particles in between to bind and unite them, resulting in the observed pores. Overall, the obtained results are representative of the experimental reference in Figure 3 (low-energy case), where the energy was insufficient to promote an increase in temperature high enough to decrease the viscosity and overcome the mobility limitation. In the simulation, there is no temperature-dependent viscosity, but a similar conclusion can be drawn since the employed viscosity values were taken at a temperature of 474 K, slightly higher than the average power bed temperature five seconds after the scan, which further proves that this viscosity is too high to promote a decent coalescence. This further justifies why the poor coalescence in the experimental results is between layers where there is less energy and the particles keep their shape and why that disappears with the temperature increase in the medium-energy case.

## 5. Conclusions and Future Work

The proposed developments in the present work make important contributions to improving the open-source computational framework for simulating the SLS process and assessing its parameter’s influence. It addresses a major limitation that hinders interactions between particles of the same material but with different viscosities by implementing an approach that utilizes an existing multi-species functionality allowing for the coalescence between used and virgin materials. This advancement significantly improves the accuracy of the solver, enabling several studies to be conducted on a more solid foundation. Building upon previous energy analysis, the cases were extended to observe the sintering evolution, and the obtained results showed a positive correlation with available experimental data, which illustrates that the developed tool can help to further improve the process. Thanks to its detailed formulation, the tool assesses the influence of each sensible physical parameter individually on essential aspects of the process, allowing for isolated studies aiming at improving specific aspects, which is difficult to achieve experimentally. The illustrated studies are a few examples of practical applications, where, for example, the dimensional accuracy and adjacent scan adhesion are evaluated with relation to laser power, scan speed and hatch distance. Moreover, with the current tool, one can also anticipate common defects related to poor coalescence and, based on the temperature profile and phase transition analysis, adapt the process to minimize them. The possible optimizations presented in this paper are a few representative applications and it is anticipated that the current framework could lead to significant advances in cases where researchers aim to optimize coalescence to minimize porosity and consequently improve mechanical properties, avoid material degradation and maximize efficiency. Furthermore, since the current tool is useful for identifying process limits, it can also be used to reverse the perspective and search for the theoretical limit instead of inputting existing parameters. This applies to material properties as well, providing guidance for future material developments. The main limitations of the current model are related to the lack of experimental data, specifically the viscosity dependency on temperature, which would further emphasize the impact of energy on coalescence. In terms of experimental validation, there is insufficient data available in the literature, and it is challenging, if not impossible, to obtain simulation-scale images during the actual process. To properly validate the results, specific tests with varying parameters need to be conducted experimentally and any potential issues should be identified. The current model is prepared to account for temperature-dependent properties, so obtaining detailed material data and additional experimental results would allow further validation.

## Figures and Tables

**Table 1:** Process and material parameters for the base case.

Parameter	Value	Units
Laser Power	17.1	W
Scan Speed	3000	mm/s
Hatch Distance	0.3	mm
Density	1000	kg/m^3^
Thermal Conductivity	0.2	W/(m K)
Viscosity (at 474 k)	390/5095	Pa·s
Surface Tension	0.035	N/m
Absorption Coefficient	1.3 × 10^4^	m^−1^
Powder Refresh Rate	50	%

## Data Availability

The raw data supporting the conclusions of this article will be made available by the authors on request.

## Abbreviations

The following abbreviations are used in this manuscript:

AM	Additive Manufacturing
SLS	Selective Laser Sintering
